# An interpretable approach to detect case law on housing and eviction issues within the HUDOC database

**DOI:** 10.1007/s10506-025-09439-9

**Published:** 2025-02-12

**Authors:** Mohammad Mohammadi, Martijn Wieling, Michel Vols

**Affiliations:** 1 Department of Legal Methods, Faculty of Law, University of Groningen, Groningen, The Netherlands; 2 Center for Language and Cognition Groningen, Faculty of Arts, University of Groningen, Groningen, The Netherlands

**Keywords:** Right to housing, Eviction, Prototype-based learning, Document classification, Interpretability

## Abstract

Case law plays a critical role in shaping our understanding of human rights, including the right to adequate housing. However, analyzing large legal databases like HUDOC, which contains over 40,000 cases, is a challenging task that requires automated solutions. This study focuses on detecting cases related to housing—a topic encompassing issues such as eviction, access to adequate housing and etc.—from the HUDOC database. For this, we developed classifiers to identify cases related to both housing and eviction issues. We first constructed a dataset using an unsupervised process refined through manual corrections. Then, we trained the Adaptive Chordal Distance-based Subspace Learning Vector Quantization models. These models achieved classification accuracies of 93% for housing-related cases and 91.5% for eviction-specific cases, matching the performance of transformer-based models while requiring fewer computational resources. Furthermore, they provide interpretability by assigning word-level importance scores, helping legal scholars understand and verify the reasoning behind the model’s predictions. The models identified 2,305 potentially housing-related cases. Manual reviews confirmed that 278 of 340 reviewed cases were indeed relevant. By detecting overlooked cases and enriching legal datasets, this study highlights the utility of NLP methods in facilitating the analysis of human rights case law. This approach supports a deeper exploration of housing rights and eviction-related decisions under the European Court of Human Rights (ECtHR), offering transparency, efficiency, and scalability for legal research.

## Introduction

The interpretation of human rights through case law is vital for understanding and applying these rights within contemporary legal frameworks Letsas (2013). Case law serves as a cornerstone in shaping the development of rights, including the right to housing, by reflecting how courts address evolving societal needs and legal complexities. As a result, legal scholars routinely rely on case law to trace the progression of human rights jurisprudence. To support this effort, judicial decisions are systematically stored in legal databases. However, the rapid growth in the volume of case law presents a significant challenge in efficiently retrieving relevant decisions, particularly in specialized areas such as housing rights. This underscores the necessity for developing automated methods to retrieve cases on the topic of interest.

With advancements in computational technology and the existence of big data, more sophisticated Natural Language Processing (NLP) models Devlin et al. (2018); Liu et al. (2019); Beltagy et al. (2020) have been developed to analyze and understand human language. These developments have expanded the application of NLP techniques within the legal domain, facilitating the automation of various tasks. For instance, topic modelling models have been used across various jurisdictions to explore various legal databases (Carter et al. 2016; Remmits 2017; Pedro Henrique and De Campos 2020; Aguiar et al. 2022; Tereza et al. 2020; Raquel Silveira et al. 2021; Razon et al. 2022; Salaün et al. 2022). Additionally, several studies have developed NLP models to classify legal documents, creating automated systems that identify, categorize, and forecast court decisions (Aletras et al. 2016; Medvedeva et al. 2020; Masha et al. 2021; Medvedeva et al. 2021, 2023). The use of large language models (LLMs) to summarize legal cases has also gained attention (Pandya 2019; Kanapala et al. 2019; Galgani et al. 2015; Kumar and Raghuveer 2012; Kim et al. 2013). Furthermore, there are works on the fine-tuning of models such as BERT using legal texts (Chalkidis et al. 2020, 2023), enhancing their understanding of specialized legal language and improving the accuracy and effectiveness of these models in legal applications.

The European Convention on Human Rights (ECHR) and the European Court of Human Rights (ECtHR) play a crucial role in interpreting and safeguarding human rights across Europe. While the ECHR establishes a framework for protecting fundamental rights, the ECtHR enforces these rights by interpreting the convention through specific cases. The HUDOC database^1^, which compiles ECtHR decisions, is an essential resource for scholars^2^. It offers a keyword search functionality that enables users to retrieve relevant cases based on specific terms. However, while keyword search can help find relevant cases, it often leads to broad and imprecise results, which may not always align with a researcher’s specific focus. This limitation creates a barrier for scholars interested in specialized areas, such as housing rights, as manual searches through vast datasets remain impractical.

A possible solution to the limitations of keyword search in HUDOC is the use of advanced NLP techniques, such as topic modeling or transformer-based classifiers. These methods aim to automate case retrieval and classification, offering a more efficient way to identify relevant legal documents. However, topic modeling, as an unsupervised approach, often extracts topics that may not align well with the topic of interest, especially for more specific issues such as eviction, leading to incomplete or irrelevant results. While supervised models, such as fine-tunning transformer-based models, can improve accuracy, their high computational costs and reliance on GPUs make them less accessible to researchers without advanced resources. As a result, despite their potential, these approaches present practical challenges for targeted case retrieval in specialized legal areas.

To address these challenges, this paper develops efficient automated models for case retrieval, focusing specifically on identifying housing and eviction-related cases within the HUDOC database. We employ the Adaptive Chordal Distance-based Subspace Learning Vector Quantization (AChorDS-LVQ) (Mohammadi and Ghosh 2024), which offers a lightweight and interpretable alternative to resource-intensive transformer models. In addition, we introduce a mechanism to convert the model’s output into a score that measures the relevance of each case to housing or eviction. Through experiments, we demonstrate the transparency and computational efficiency of the resulting models, showcasing their ability to explain decisions and operate without the need for GPU. We further illustrate how the relevance scores help prioritize the most relevant cases, making the retrieval process more efficient, especially when manual annotation resources are limited. Ultimately, the models generate a list of cases likely related to housing and eviction, contributing to a more comprehensive dataset. This advancement facilitates more accurate and extensive empirical analysis for legal scholars, particularly in specialized areas like housing rights.

This contribution is organized as follows: Section 2 explains how we collect the required data for training models. Section 3 provides a brief introduction to the AChorDS-LVQ. Section 4 covers the experiments where we developed models for case law classification and then used them to detect more relevant cases. We finally end with a conclusion in Sec. 5.

## Data

This paper aims to automate the process of detecting housing and eviction-related cases within the case law of the European Court of Human Rights (ECtHR). To achieve this, we collected all cases (judgments and decisions) available in the HUDOC database, published up to and including January 2023, for which English texts are provided. This initial collection resulted in a total of 41,621 cases. Given the significance of Article 8 (right to respect for private, family life, home and correspondence) and Article 1 of Protocol No. 1 (right to protection of property) of the European Convention on Human Rights (ECHR) in housing-related matters (Bruijn and van Tongeren 2024), we specifically targeted cases citing these articles. This filtering process yielded 13,343 cases that formed the basis for constructing a dataset aimed at developing an automated system.

Annotating all 13,343 cases manually is impractical. Therefore, we used an unsupervised approach to detect housing-related cases. We applied a step-by-step process that grouped cases based on their topics and citation patterns. First, we used topic modeling to identify the main themes within each case. This allowed us to represent each case as a topic vector. Next, we created a citation network based on metadata from HUDOC and assigned scores to each citation by examining how similar the topics of cited cases were to each other. This resulted in a weighted citation network. To cluster the cases into groups with similar themes, we applied the Louvain algorithm, a community detection method^3^. This approach grouped cases that addressed similar issues, resulting in clusters that were more internally consistent. From these clusters, we selected the ones where most cases dealt with housing issues. This process narrowed down the dataset to 1,108 cases that were likely to be housing-related.

Following pre-selection, each case was manually annotated by a team of four trained annotators to ensure consistent evaluation of relevance to housing issues. The annotation process focused on examining the facts of the case and the applicant’s complaints. A case was classified as housing-related if the facts or complaints predominantly concerned the someone’s house, home, residence, or accommodation. Additionally, cases involving disputes between landlords and tenants were categorized as housing-related. For eviction-specific classification, the annotators applied a broad, tenure-neutral definition of eviction, encompassing the loss of a home regardless of the applicant’s legal status (e.g., tenant, homeowner, squatter, or illegal occupier).

To standardize the annotation process, annotators were provided with a comprehensive codebook outlining definitions, examples, and decision criteria. The annotators underwent training sessions, during which they reviewed and discussed sample cases to align their interpretations. Inter-annotator agreement was measured using Fleiss’ kappa score, which ranged from 0.81 to 0.89, indicating (almost) perfect agreement (0.81-1.00). This high level of agreement underscores the reliability of the annotation process and the consistency of the classification outcomes.

As expected, the majority (78%) of the 1,108 cases in our manually annotated dataset were housing-related, resulting in an imbalanced dataset. This can lead to bias in the model toward predicting cases as housing-related. To reduce this effect, we randomly selected 507 additional cases linked to Article 8 ECHR or Article 1 of Protocol No. 1 ECHR that were not among the 1,108 cases. A summary of the resulting dataset is presented in Table 1. While the majority of the new cases are indeed unrelated to housing issues, there are still cases on housing that were not identified previously (12%). This shows that unsupervised methods, such as those used to collect the initial 1,108 cases, may miss relevant cases. Since it groups cases by topics and citation patterns, it tends to overlook cases that do not strongly cite housing-related cases. This highlights the need for supervised models to identify housing-related cases based solely on the text, rather than citations, by training the model with examples that clearly represent housing (and eviction)-related cases.

**Table 1:** Summary statistics of the collected dataset

Method	Unsupervised	Random	Total
Housing	865	63	928
Not-housing	243	444	687
Eviction	678	26	704
Not-eviction	430	481	911
Total	1108	507	

That the eviction cases consists of a subset of the housing cases

We aim to utilize this manually annotated dataset of case law to build classifiers that predict case law labels, specifically focusing on identifying those related to housing and eviction. This dataset, which includes cases linked to both Article 8 and Article 1 of Protocol 1 of the ECHR, will be split into training (80%) and testing (20%) sets. Thus, both sets will include cases related to both articles, ensuring that the classifier is trained and evaluated on cases from the full range of relevant legal provisions.

## Method

In this section, we present an overview of the Adaptive Chordal Distance-based Subspace Learning Vector Quantization (AChorDS-LVQ) method, which we use to classify case law. To achieve this, we begin with a brief introduction to the Generalized Learning Vector Quantization (GLVQ) framework, the foundation for the AChorDS-LVQ method. We then give a summary of AChorDS-LVQ which combines the power of word embedding models with the interpretability of GLVQ, offering a robust solution for applications that require both accuracy and interpretability.

### Generalized learning vector quantization

Generalized Learning Vector Quantization (GLVQ) Sato and Yamada (1995) represents a family of classifiers known for their intrinsic ability to explain their decisions. It utilizes a set of labelled prototypes, which are representative examples summarizing the characteristics of each class. Because of its transparency, it has been applied in different fields where it is necessary to have a transparent model, ranging from healthcare (Ghosh et al. 2020, 2022; Mohammadi et al. 2017) and education (Widiantara et al. 2023), to astronomy (Mohammadi et al. 2022).

**Fig. 1:**
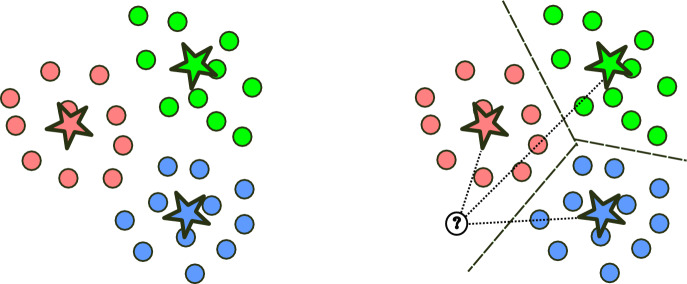
A conceptual visualization of LVQ classifiers. Left: training examples (circles) and learned prototypes (stars) with colours as labels. Right: illustration of the Nearest Prototype Classifier (NPC) strategy with an uncoloured circle as an unseen example. Note that the dashed lines represent borders between classes and dotted lines denote the difference between an unseen example and prototypes

Let $\{ (\vec {x}_i, y_i) \}_{i=1}^N$ be the training set where $\vec {x}_i \in \textrm{R}^D$ is a training example and $y_i$ is its corresponding label. Given the training set, a GLVQ classifier models classes through a set of labelled prototypes, i.e.:

$$\begin{aligned} \Big \{ \big (\vec {w}_i, c(\vec {w}_i)\big ) \Big \}_{i=1}^p \end{aligned}$$

where $\vec {w}_i \in \textrm{R}^D$ denotes a prototype vector and $c(\vec {w}_i)$ is its corresponding label. As can be seen in the left visualization presented in Fig. 1, prototypes capture major characteristics of each class and their distribution simulates the distribution of classes within the data. During training, the classifier learns the best places for prototypes by optimizing the following cost function (more details in Sato and Yamada (1995)).

1 $$\begin{aligned} E = \sum _{i=1}^N E_i = \sum _{i=1}^N \text {sgd} \Big ( \frac{d(\vec {x}_i, \vec {w}^+) - d(\vec {x}_i, \vec {w}^-)}{d(\vec {x}_i, \vec {w}^+) + d(\vec {x}_i, \vec {w}^-)} \Big ) \hspace{5.0pt}, \end{aligned}$$

where sgd is the sigmoid function, and *d*(., .) represents a distance measuring the difference between an example and a prototype (dotted lines in Fig. 1, right). Note that one can interpret $E_i$ as the probability of misclassifying the i-th example. Consequently, the model aims to reduce the overall probability of misclassifications.

Once the model learns the optimal prototypes, it follows the Nearest Prototype Classifier (NPC) strategy to perform predictions for unseen examples (see Fig. 1, right). This means that for a new example $\vec {x}$, we first find the closest prototype to it. Once we find the closest prototype, we assign its label (red colour in the example shown in Figure 1, right) to $\vec {x}$. In the context of case law, it will classify a case as housing related, only if it’s closest to the prototype representing housing-related cases (and not another prototype).

Since prototypes are positioned within the data space ($\mathbb {R}^D$), so the complete set of cases, they are highly iterpretable and one can easily identify the major characteristics within classes (e.g., through shared concepts). Due to GLVQ’s explanatory power, different variants of it have been developed to enhance its flexibility and interpretability. These variants, including AChorDS-LVQ (Mohammadi and Ghosh 2024), differ by proposing different distance measures *d*.

### Adaptive chordal distance-based subspace learning vector quantization

The first step towards the classification of textual data such as case law of the ECtHR is to convert the textual data to a numerical representation. In other words, we need to transform words into numbers such that these numbers capture the meaning of the words. In simpler terms, if two words have similar meanings, their numerical representations (i.e. two series of numbers contained in two vectors) also need to be close to each other. With the advancement in word embedding techniques, several approaches have been developed to capture the semantic meaning of words in numerical vectors. This has started with Word2Vec (Mikolov et al. 2013) and GloVe (Pennington et al. 2014) which use the co-occurrence of words to determine similarity (e.g., ‘cat’ will more often co-occur with ‘dog’ than with ‘judge’, and therefore the vector representations of ‘cat’ and ‘dog’ will be more similar than that of ‘cat’ and ‘judge’). These approaches therefore are an efficient and effective way to represent words and text through numbers.

Let $\text {doc} = (\text {word}_1, \text {word}_2, \cdots , \text {word}_n)$ be a text with *n* words. A word embedding model assigns a vector representation to each word, resulting in the text being represented as a set of vectors:

$$\begin{aligned} (\text {word}_1, \text {word}_2, \cdots , \text {word}_n) \xrightarrow []{\text {GloVe}} (v_1, v_2, \cdots , v_n) \end{aligned}$$

where $v_i$ is the numerical representation for the i-th word $\text {word}_i$, called an embedding vector.

The conventional machine learning approaches, such as Support Vector Machines (SVM) and GLVQ, accept one vector per example as input. To apply these methods to textual data, it is therefore necessary to summarize an entire text into a single vector instead of a set of vectors. A common practice to prepare data is to use the mean vector, i.e. $\frac{1}{n} \sum _{i=1}^n v_i$. However, this summarization can result in losing valuable information needed to differentiate documents effectively.

To address this issue, AChorDS-LVQ (Mohammadi and Ghosh 2024) instead uses a set of vectors to represent a text. This approach aligns better with the nature of texts, which consist of many words rather than a single word, thus preserving more information and improving classification accuracy. Since the number of words *n* in documents can vary between documents, AChorDS-LVQ employs a dimension reduction technique, Singular Value Decomposition (SVD), to represent each document with a fixed number of vectors *d*^4^:

$$\begin{aligned} (\text {word}_1, \text {word}_2, \cdots , \text {word}_n) \xrightarrow []{\text {GloVe}} (v_1, v_2, \cdots , v_n) \xrightarrow []{\text {SVD}} (u_1, u_2, \cdots , u_d) \end{aligned}$$

Being a member of the GLVQ family, AChorDS-LVQ defines a set of labelled prototypes $\{(W_i, y_i)\}_{i=1}^p$, where each prototype $W_i$ consists of *d* vectors, instead of a single vector. This flexibility, achieved by using multiple vectors for both prototypes and documents, enables the model to achieve document classification results comparable to transformer-based models like BERT and RoBERTa (Mohammadi and Ghosh 2024). However, it does so with less computational power (Mohammadi and Ghosh 2024). More importantly, AChorDS-LVQ offers transparency in its decision-making process. It assigns a score to each word representing its influence on the final decision. Positive scores indicate words that contribute to the model’s prediction in favour of the predicted label, while negative scores reflect words that argue against the predicted label, suggesting they reduce the likelihood of the predicted classification (see Mohammadi and Ghosh (2024) for more details). This transparency makes the model’s reasoning more interpretable, allowing users to directly examine which words have the greatest impact on the classification decision. In this way, AChorDS-LVQ not only offers comparable performance to more complex models but also provides valuable insights into how much each word influences the outcome, aligning the model’s logic with human expectations.

## Experiment

In this section, we develop classifiers to identify cases addressing housing and eviction issues. The section is structured as follows: First, we describe the pre-processing steps applied to prepare the dataset in formats suitable for machine learning models. Next, we construct and evaluate a range of classification models using the annotated dataset to determine whether a given case concerns housing (or eviction) issues. Finally, we apply the trained models to classify cases not included in our initial annotated dataset. This allows us to identify potential new housing-related cases that were not captured during the initial dataset construction phase.

### Experimental setup

Our goal is to develop binary classifiers to label case law documents as either not addressing a housing-related issue (0), or addressing a housing-related issue (1). Similarly, we create binary classifiers that label case law documents as eviction-related. In order to generate the training and test sets, we randomly select 80% of the cases in our manually annotated dataset as training examples, and we use the rest of the dataset to evaluate our approach. This results in 1,292 cases used for training (728 on housing and 561 on evictions) and 323 cases for testing (200 on housing and 143 on evictions).

Given the limited amount of annotated data available (fewer than 2000 cases), we use two strategies for building our classifiers:

- Learning from scratch: this approach involves training models from the ground up using our annotated dataset. However, due to the small size dataset, only models with lower complexity are feasible, such as SVM (Hearst et al. 1998), GRLGQ (Mohammadi et al. 2024) and AChorDS-LVQ (Mohammadi and Ghosh 2024).
- Fine-tunning Large Language Models (LLM): Through technological advances, we have access to more complex models with millions (or billions) of parameters. These models are trained on large corpora of texts, seemingly obtaining somewhat of an understanding of the structure of human language. These models can subsequently be fine-tuned with smaller datasets for specific tasks, such as in this case classifying legal documents.

In this study, we trained SVM (Hearst et al. 1998), GRLGQ (Mohammadi et al. 2024) and AChorDS-LVQ (Mohammadi and Ghosh 2024) models from scratch. Additionally, we fine-tuned three large pre-trained models: (a) Bidirectional Encoder Representations from Transformers (BERT) (Devlin et al. 2018) pre-trained on general texts, (b) Legal-BERT (Chalkidis et al. 2020) pre-trained on legal data, and (c) Longformer (Beltagy et al. 2020) pre-trained on general texts but designed to accept longer texts.

To train our models, we first need to prepare the documents appropriately. For simpler models, we must convert the text into numerical representations. A common method for this is to use word embedding models, such as Word2Vec and GloVe. Following the approach outlined in Mohammadi and Ghosh (2024), we use the GloVe model to represent the text. The preprocessing steps are as follows:

1. *Stop words removal* We begin by removing all stop words (i.e. uninformative function words), such as ‘and’ and ‘is’, to reduce the influence of common words that add little value to the document’s meaning.
2. *Word embedding* Using the GloVe model, we generate a vector representation for each word in the document.
3. *Case law representation*
  *SVM* We represent each document by calculating the mean vector of all the word vectors generated by GloVe.
  *GRLGQ and AChorDS-LVQ* For these models, we apply Singular Value Decomposition (SVD) to the word vectors to generate the appropriate document representation with multiple vectors.

This process generates the required input for SVM, GRLGQ and AChorDS-LVQ.

Due to the computational complexity, transformer-based language models, such as BERT, have a limit on the length of text they can process, known as the context window. For instance, BERT and LegalBERT are restricted to a maximum of 512 tokens. There are several approaches to address this limitation. A common approach to tackle this challenge is to segment the long text into smaller pieces and then perform prediction for each piece separately. Following that approach, we split documents here into non-overlapping chunks of 300 words. During training, we assign to each chunk the document’s label and then use it to train BERT and LegalBERT. At test time, the model predicts labels for each chunk individually, and then the document’s predicted label is the one that appears most frequently among its corresponding chunks. When we report the results, we add the subscript ‘long’ to the model names, to distinguish this adjustment.

Another approach to address the limited context window is to find a way to reduce the computational cost. For this, the Longformer model has been introduced (Beltagy et al. 2020). This model can handle documents with 4096 tokens. As more than 50% of documents in our dataset are shorter than 2300 words, we directly use Longformer on our data without any splitting.

### Model performance on the test set

Table 2 summarizes the accuracies of various approaches for classifying case law as either housing-related or eviction-related. For housing classification, the AChorDS-LVQ and GRLVQ models achieve comparable results to transformer-based models, with AChorDS-LVQ yielding the best accuracy. Notably, AChorDS-LVQ accomplishes this with significantly fewer parameters (30k) compared to the transformer-based models such as LegalBERT$_\text {long}$, which has 110M parameters. This demonstrates the computational efficiency of AChorDS-LVQ and GRLVQ, making them particularly suitable for large-scale applications.

**Table 2:** Accuracies of different methods on both binary classification tasks

Method	# of para.	Housing	Eviction
SVM	256k, 260k	87.62	82.97
GRLGQ	30k	93.11 ($\pm 0.11$)	90.92 ($\pm 0.15$)
AChorDS-LVQ	30k	93.60 ($\pm 0.15$)	91.53 ($\pm 0.39$)
BERT	110M	91.33	87.62
LegalBERT	110M	92.57	88.85
BERT$_\text {long}$	110M	93.17	91.64
LegalBERT$_\text {long}$	110M	93.48	92.24
Longformer	148M	93.19	91.64

For the eviction classifier, all models achieve a lower performance compared to the housing classifiers. This may arise from the inherent differences between the two classes. Specifically, the eviction class represents a highly specific legal issue, whereas the non-eviction class comprises a broader range of topics, including other housing-related disputes (e.g., occupancy or entrance issues). This diversity within the non-eviction class makes it harder for models to distinguish eviction-specific cases, as more nuanced information may be required to separate them from other housing-related cases.

In this context, LegalBERT$_\text {long}$ outperforms the others. One possible reason for this is that LegalBERT, which was pre-trained on legal data, already has a general understanding of legal texts, enabling it to achieve a higher accuracy even with small datasets. Despite providing slightly lower accuracy, AChorDS-LVQ still delivers acceptable results, offering a good balance between accuracy and computational cost.

As previously mentioned, AChorDS-LVQ is a transparent model that assigns an importance score to each word in a case law document. These scores quantify the influence of individual words on the model’s final decision. A higher score indicates a stronger positive contribution of a word toward the predicted label, while a lower score reflects a negative contribution, arguing against the predicted label. In essence, words with small negative scores provide reasons against the predicted classification. This feature allows users to understand the reasoning behind the model’s predictions and offers valuable insights into the underlying decision-making process (for additional details, see Mohammadi and Ghosh (2024)).

**Fig. 2:**
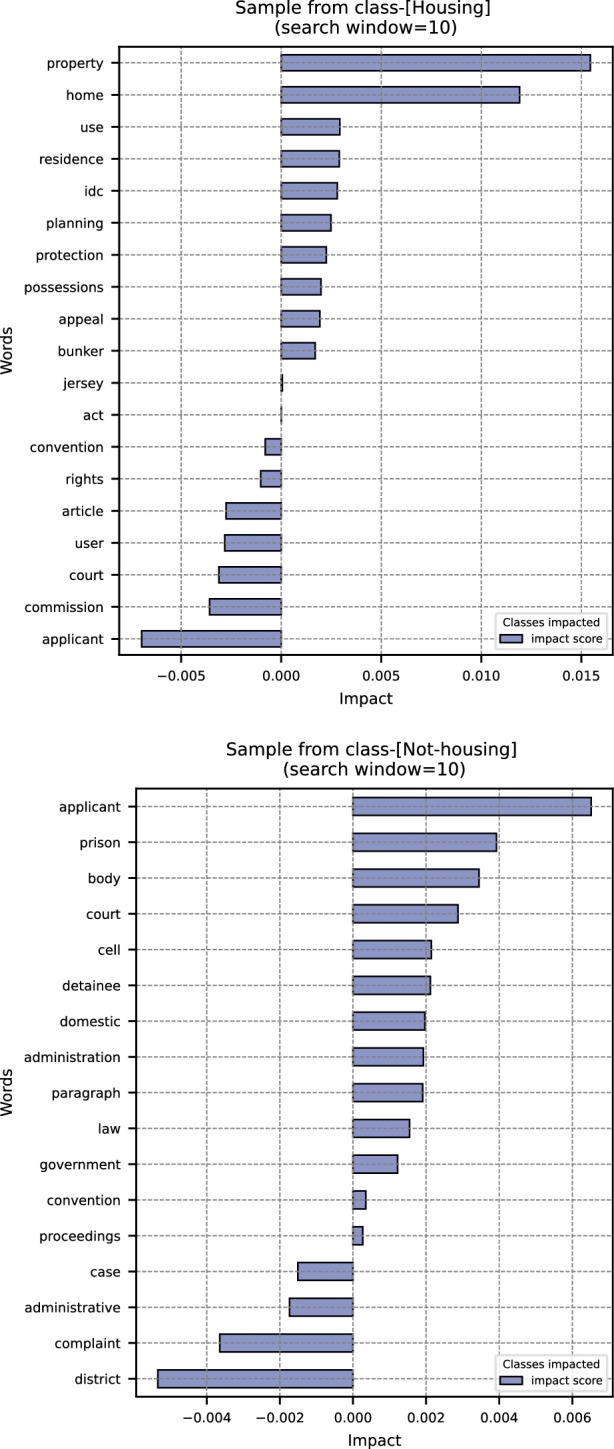
Top words with their impact on the classifier’s decision for *Muriel Herrick v. the United Kingdom* (a housing-related case). Bottom: *Merzalijevs v. LATVIA* (a non-housing case)

To demonstrate this interpretability, we present examples from each class, highlighting the words that have the greatest impact on the model’s decisions. Specifically, we applied the model to the *Muriel Herrick v. the United Kingdom*^5^. The model correctly predicts that the case involves a housing-related issue, and it also assigns a numerical value to each word, indicating its influence on our model’s decision. The above graph shown in Fig. 2 displays several words with the highest impact. The blue bar represents the extent to which a word supports the correct class (i.e. ‘housing’). The larger (i.e. more towards the right) the blue bar, the more important the word is for the correct class ‘housing’. Conversely, the smaller (i.e. more towards the left) the blue bar, the more important the word is for the incorrect class ‘non-housing’. It can be seen that words such as ‘property’, ‘home’, ‘residence’, and ‘bunker’ are important for housing-related cases, aligning well with our intuition. Additionally, we observe that while housing-related terms correctly support the claim that the case covers a housing issue, procedural terms such as ‘convention’, ‘article’, and ‘court’ do not.

Similarly, we utilized the model to analyze the ECtHR’s decision *Merzalijevs v. Latvia*^6^. The model correctly classifies this case as non-housing-related. The bottom graph shown in Fig. 2 highlights the most important words influencing this prediction. For this particular case, words such as ‘prison’, ‘cell’ and ‘detainee’ are highlighted by the model. These terms suggest that this case is related to prison conditions, thereby reinforcing the model’s classification. This detailed analysis confirms the model’s capability to accurately interpret and classify case law based on the specific terminology used.

**Fig. 3:**
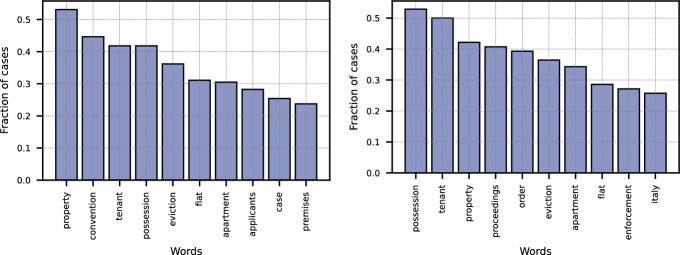
Fraction of cases containing the 10 words most frequently identified as important by the AChorDS-LVQ models: Left) for housing class, and Right) for eviction class

To gain deeper insights into the prototypes and the patterns they capture, we analyzed the model’s predictions across the entire housing-related cases in the test set. For each case, we extracted the 10 most influential words identified by the AChorDS-LVQ model. Fig. 3 left illustrates the fraction of cases in which each of them was identified as significant. The results show that words such as ‘property’, ‘tenant’, ‘eviction’, ‘flat’, and ‘apartment’ were frequently highlighted by the model as important for its predictions. This aligns closely with our expectations for a model trained to identify housing-related cases, as these terms are intrinsically linked to housing issues. Similarly, Fig. 3 right displays the words most frequently identified as important by the eviction-specific model. The overlap between the two models is evident, with both highlighting terms such as ‘tenant’, ‘flat’, ‘eviction’, and ‘property’, reflecting the significant presence of eviction cases within the broader housing-related set (76% of housing-related cases are eviction-related). However, for the eviction-specific model, the fraction of terms like ‘tenant’, ‘flat’, and ‘possession’ is higher compared to the housing model. This suggests that the eviction model places a stronger emphasis on terms more specific to eviction cases, further reinforcing the distinction between the two types of housing-related issues.

#### Discussion

The results demonstrate the practicality and cost-effectiveness of AChorDS-LVQ for detecting housing and eviction-related cases in the ECtHR database. Its computational efficiency, stemming from a lightweight architecture with only 30k parameters, makes it an accessible option for scenarios with limited computational resources. Unlike transformer-based models such as Legal-BERT$_\text {long}$, which require GPU resources for training, AChorDS-LVQ can be trained on standard CPUs, reducing costs and increasing scalability for real-world applications (see 6 for details).

The interpretability of AChorDS-LVQ is another important advantage. By identifying the most influential words for each prediction, the model provides insights into its decision-making process. This transparency is particularly valuable in legal contexts, where scholars need to understand and trust automated systems. By analyzing the highlighted words, we observed that the housing and eviction-related models frequently identified terms such as ‘property’, ‘tenant’, ‘flat’, and ‘apartment’, reflecting the model’s alignment with human expectations. This demonstrates that the learned patterns in both models are well-aligned with legal reasoning, reinforcing the reliability and interpretability of the model’s predictions.

The lower performance of the eviction classifier highlights the challenges of distinguishing narrowly defined categories from more general ones. While the dataset is (almost) balanced, the diversity of the non-eviction class introduces noise, making it harder to identify eviction-specific features.

The ability of AChorDS-LVQ to combine interpretability with competitive performance and low resource requirements positions it as a robust solution for legal text classification. Thus, we have opted to use AChorDS-LVQ in our analysis for the following sections.

### Detection of new housing-related cases

In this section, we explore the performance of the trained classifier, which was developed to distinguish between housing-related and non-housing-related cases, on a set of 41,621 unannotated cases. Given that our training and testing sets only cover cases related to Article 8 and Article 1 of Protocol No. 1, we decided to examine the classifier’s performance in two distinct scenarios. First, we evaluate the model’s predictions on cases involving Article 8 and Article 1 of Protocol No. 1. This analysis aims to assess the generalizability of the model within the same legal framework on which it was trained. Second, we extend the evaluation to a broader set of 28,278 cases, which involve other articles not included in the training set. This scenario allows us to investigate how well the model generalizes to entirely new legal contexts beyond those seen during training. These two subsections will provide insights into both the model’s ability to generalize within its original scope (Article 8 and Article 1 of Protocol No. 1) and its capacity to handle cases from previously unseen articles.

#### Detection of housing-related cases under Article 8 and Article 1 of Protocol No. 1

The HUDOC database has 13,343 cases addressing Article 8 or Article 1 of Protocol 1 ECHR. As indicated, we have manually coded 1,615 cases of these cases (12%). To identify other housing-related cases, we applied the same model, used in the previous section, to all these cases. This resulted in the detection of 1,738 cases potentially related to housing issues. Given the large number of newly detected cases and the time-consuming process of manual annotation, it may be useful to prioritize the cases with the highest likelihood of being housing-related. Therefore, we developed a way to convert the model output into probability scores. These scores represent the likelihood that a given case is related to housing, allowing us to rank the cases accordingly. As a result, we can focus more effectively on the most relevant cases.

Following the definition of the cost function in Equation 1, we use the calculated distances to prototypes to define the probability of a case being related to housing as follows:

2 $$\begin{aligned} P(\text {case is on housing}) = \text {sgd} \left( \frac{d(\vec {x_i}, \vec {w}^{\text {NH}}) - d(\vec {x_i}, \vec {w}^{\text {H}})}{d(\vec {x_i}, \vec {w}^{\text {NH}}) + d(\vec {x_i}, \vec {w}^{\text {H}})}\right) \end{aligned}$$

where $d(\vec {x_i}, \vec {w}^{\text {NH}})$ and $d(\vec {x_i}, \vec {w}^{\text {H}})$ represent the distance between the document $\vec {x}_i$ and the prototypes of non-housing (NH) and housing (H), respectively.

**Fig. 4:**
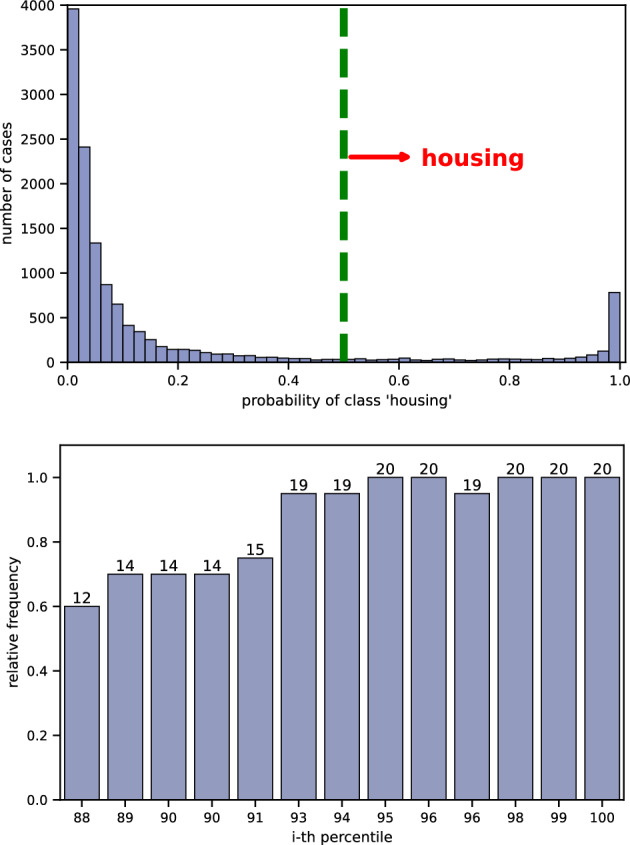
Results for cases involving Article 8 and Article 1 of Protocol 1. Top: Distribution of probability scores (as defined in Eq. 2) indicating the likelihood of a case being housing-related. Bottom: Fraction of manually annotated cases from the top 13 percentiles that were confirmed to be housing-related.

The top graph in Fig. 4 shows the distribution of probability scores of being housing-related (from Eq. 2). As expected, most cases receive low scores, indicating they are unlikely to be related to housing. Using these scores, we can set a threshold to identify cases that are more likely to be housing-related. By default, the model predicts a case as housing-related if its score is above 0.5 (on the right side of the green line).

To determine the best threshold, we calculated the percentile for each score. We found that the 88th percentile corresponds to a score of 0.502. To further assess the threshold, we randomly selected and manually annotated 20 cases from each of the top 13 percentiles. The bottom graph in Fig. 4 shows the results of this manual check. These results clearly show a direct link between the scores and the likelihood of a case being housing-related. A higher threshold means most of the selected cases are more likely to be housing-related, while a lower threshold could include more non-housing cases. For example, setting the threshold at 0.91 (93rd percentile) would result in cases that are over 95% (19 out 20) likely to be housing-related. However, choosing a lower threshold will result in more cases being selected, so the threshold should be adjusted based on how many human annotators are available to check the cases.

In summary, this analysis highlights the model’s effectiveness in uncovering relevant cases that might have otherwise been overlooked. Consequently, this method may help in providing a more accurate and thorough understanding of the application and interpretation of Article 8 and Article 1 of Protocol 1 in housing-related matters as it can more completely identify housing-related cases.

#### Detection of housing-related cases from other articles

Although Article 8 and Article 1 of Protocol No. 1 of the European Convention on Human Rights (ECHR) are typically the primary articles addressing housing-related issues, other articles may also involve housing matters. To expand the scope of our housing-related dataset, we applied the model to the remaining cases in the HUDOC database, which contains 28,278 cases, with the goal of detecting housing-related cases under different articles. The trained model identified 567 cases with probability scores greater than 0.5, suggesting that they are likely related to housing.

**Fig. 5:**
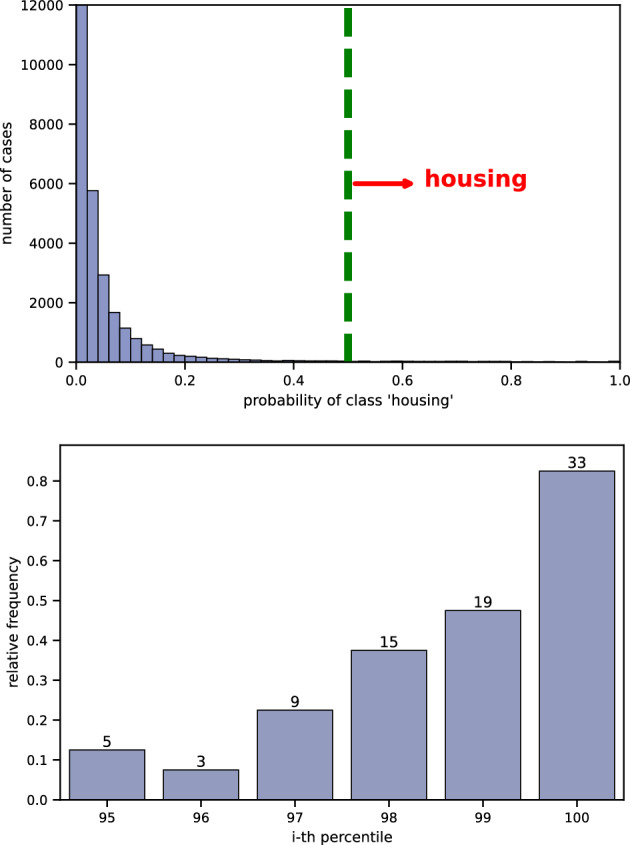
Results for cases not related to Article 8 and Article 1 of Protocol 1. Top: Distribution of probability scores (as defined in Eq. 2) indicating the likelihood of a case being housing-related. Bottom: Fraction of manually annotated cases from the top six percentiles confirmed to be housing-related. Note that only the last two percentiles (and a small portion of the third-last percentile) were predicted as housing-related by the model

Similar to the previous experiment, we calculated the probability scores (from Eq. 2) representing the likelihood of cases being related to housing. The top graph shown in Fig. 5 illustrates the distribution of these scores. The majority of cases receive low scores, with only the top two percentiles receiving scores above 0.5.

To evaluate the model’s performance across different score ranges, we randomly selected and annotated 40 cases from each of the top six percentiles (i.e., cases with scores above 0.21)^7^. The bottom graph in Fig. 5 presents the results of this manual annotation. Here, the model’s performance is lower for these cases compared to those under Article 8 and Article 1 of Protocol No. 1. The performance drop results from a domain shift, as cases from other articles differ in textual structure and legal context compared to the primary training data. Nevertheless, the model is still useful in detecting housing-related cases from other articles that might have been missed otherwise. For instance, when applying a higher threshold (e.g., 0.71), the model predicts that 82.5% of the cases identified are housing-related.

We also observe that as the probability score decreases, the likelihood that a case is housing-related also tends to decrease. For example, a case with a score of 0.52 has a 47.5% chance of being housing-related, whereas a case with a score of 0.25 has only a 7.5% chance. Furthermore, the percentage of correctly identified housing-related cases drops sharply from 82.5% (for scores above 0.71) to 47.5% (for scores between 0.52 and 0.71). This demonstrates the importance of setting a higher threshold to focus on the cases the model is most confident about (i.e., above 0.71). However, cases with lower scores can still be reviewed if human annotators are available, as the probability of identifying housing-related cases remains greater than zero, even at lower score percentiles.

This experiment shows the utility of the model in identifying and prioritizing housing-related cases across various legal articles. It enables legal researchers to uncover housing-related judicial decisions beyond the primary housing-related articles, providing a more comprehensive view of housing law and judicial interpretation.

## Conclusion

As international human rights conventions are considered ‘living instruments’, legal scholars must continuously track the developments of conventions to enhance our understanding of human rights. In this context, empirical research on court decisions plays an important role in our understanding of the interpretation and application of these conventions. However, this task is challenging given the large legal databases, and it is therefore necessary to be able to automatically identify relevant case law on specific issues. In this study, we developed and evaluated models to retrieve housing and eviction-related cases from the HUDOC database. Our goal was to enhance the retrieval of relevant cases, thereby assisting legal scholars in analyzing housing rights within the European Convention on Human Rights (ECHR).

We addressed two classification tasks: a general classification task focusing on the right to housing (including aspects such as adequate housing, eviction and etc.) and a more specific classification task centered solely on eviction cases. To achieve this, we first constructed a dataset on housing-related issues using an unsupervised process that was manually corrected and extended. While the unsupervised approach provided an initial baseline, it had limitations, particularly its tendency to miss housing-related cases that lack strong connections (e.g., citations) to other housing-related cases. By applying AChorDS-LVQ, we achieved a significant improvement in accuracy, increasing it from 78% (unsupervised) to 93%, thereby demonstrating the effectiveness of this model in this application.

Through our experiments, we demonstrated that AChorDS-LVQ offers a unique balance of computational efficiency and interpretability. Unlike transformer based models, such as BERT and Longformer, AChorDS-LVQ operates with significantly lower computational requirements, enabling it to run on CPUs rather than GPUs. This reduces the computational cost and facilitates regular updates with new data. Additionally, its interpretability is a key advantage: the model assigns importance scores to individual words, quantifying their influence on the classification decision. This transparency allows users to understand why specific cases are classified as housing-related, making the model’s predictions more reliable and interpretable for legal scholars.

We then applied our models to the unannotated data within the HUDOC database, resulting in the detection of many new cases related to housing issues. Specifically, the model detected 1,738 potentially housing-related cases under Article 8 and Article 1 of Protocol No. 1. A manual review of 260 of these cases confirmed that 226 (87%) were indeed housing-related. Similarly, 567 cases were identified from other articles, with a manual review of 80 cases confirming 52 (65%) as relevant. To enhance efficiency, we introduced a scoring system based on the model’s outputs to prioritize cases by their likelihood of relevance. Our experiments demonstrated that this system enables a more effective allocation of resources, allowing the most relevant cases to be reviewed and annotated first.

This study highlights the potential of AChorDS-LVQ to enhance the identification of housing-related cases within large legal databases. By detecting relevant cases both within and beyond the articles typically associated with housing rights (i.e., Article 8 and Article 1 of Protocol No. 1), our approach provides a more comprehensive understanding of how the ECHR interprets the right to housing. To further improve generalizability and address the challenges posed by domain shifts (for cases covering other articles), future work will focus on constructing a more diverse training dataset that encompasses a broader range of legal contexts and textual structures. By doing so, we aim to refine the model’s ability to accurately classify housing-related cases across diverse scenarios. With its computational efficiency, transparency, and high accuracy, AChorDS-LVQ demonstrates its utility as a powerful tool to support legal scholars in their analysis of evolving housing rights in international law.

## Appendix: Model specifications and computing resources

See Table 3.

**Table 3:** The performance of different classifiers on document classification task

Model	Version and documentation link	Computing resource used
BERT	google-bert/bert-base-uncased	T4GPU (via Google Collaboratory)
LegalBERT	nlpaueb/legal-bert-base-uncased	T4GPU (via Google Collaboratory)
Longformer	allenai/longformer-base-4096	A100GPU (via Google Collaboratory)
SVM	Sklearn SVM	CPU
GRLGQ	Code repository of GRLGQ in Github	CPU
AChorDS-LVQ	Proposed method in this contribution. Code repository will be made public after acceptance of this work.	CPU
word2vec	word2vec-google-news-30	
GloVe	glove.42B.300d	

## Footnotes

1 https://www.echr.coe.int/hudoc-database.

2 Note that from 18 October 2022, the Court’s Knowledge Sharing platform (ECHR-KS) is publicly available (https://www.echr.coe.int/hudoc-database). It aims to share Convention case-law knowledge.

3 Mohammadi et al. (2024).

4 Note that the application of SVD not only allows for representing documents in a fixed-size matrix, but it also serves as a dimensionality reduction technique that filters out noise (i.e., less useful information) and retains the most important features of the data. This ensures that the model focuses on meaningful patterns, improving both its robustness and interpretability.

5 ECHR 11 March 1985, no. 11185/84 (*Muriel Herrick v. the United Kingdom*).

6 ECHR 13 November 2014, no. 1088/10 (*Merzalijevs v. Latvia*).

7 Note that only the last two percentile are predicted as housing-related by the model.
